# Linking the Results of CIPM and RMO Key Comparisons With Linear Trends

**DOI:** 10.6028/jres.115.010

**Published:** 2010-06-01

**Authors:** Nien Fan Zhang

**Affiliations:** National Institute of Standards and Technology, Gaithersburg, MD 20899

**Keywords:** degrees of equivalence, generalized least squares estimator, key comparison reference value, linking laboratory, uncertainty

## Abstract

A statistical approach to link the results of interlaboratory comparisons with linear trends is proposed. This approach can be applied to the case that the comparison artifacts have the same nominal values or the measured quantities have the same magnitudes. The degrees of equivalence between the pairs of National Metrology Institutes that have not participated in the same comparisons, and their corresponding uncertainties are established. The approach is applied to link the CCEM-K2 and SIM.EM-K2 comparisons for resistance at 1 G Ω level.

## 1. Introduction

The work of linking the results of International Committee for Weights and Measures (CIPM) and Regional Metrology Organization (RMO) key comparisons (KCs) is an important part of implementing the CIPM Mutual Recognition Arrangement (CIPM MRA) of the CIPM. Recently, several methodologies have been proposed to deal with the linkage problem. Delahaye and Witt [1] proposed a practical method, which used an additive correction to link a CIPM KC of 10 pF capacitance standards to results obtained by a corresponding EUROMET comparison. A similar method was used to link key comparisons CCEM-K8 and EUROMET.EM.K8 by Marullo Reedtz and Cerri [2]. Elster, Link, and Wöger [3] suggested a method based on a ratio correction, which can be applied when the results of the CIPM and the RMO comparisons are of different magnitude or different physical dimension. Nielsen [4] and Sutton [5] suggested combining the measurements from CIPM and RMO key comparisons by applying weighted least squares or generalized least-squares estimation. As pointed out in [3], however, this approach will generate a completely new analysis, which obviously will influence the existing results. Kharitonov and Chunovkina [6] and Decker et al. [7] have also discussed linking of CIPM and RMO key comparisons.

Zhang et al. [8] proposed a statistical approach to KCs with linear trends. Later, Zhang et al. [9] extended the results to the case of multiple artifacts. Discussions of key comparisons with trends can also be found in [10] and [11]. In this paper we propose a method to link the existing results from CIPM and RMO KCs both of which have linear trends.

Section 2 provides the statistical models and major results for key comparisons with linear trends based on the general case discussed in [12]. In Sec. 3, the difference between the degrees of equivalence of the two comparisons is defined and used to establish the relationship between these two comparisons. An estimator of this quantity is proposed, and is used to estimate the degree of equivalence of a laboratory that participated only in the RMO KC, with respect to the key comparison reference value (KCRV) of the corresponding CIPM KC. In Sec. 4, degrees of equivalence with their corresponding uncertainties, are established between pairs of National Metrology Institutes (NMIs) that only participated in one of the two comparisons. In this study we assume that the artifacts in the two KCs have the same nominal values or values of the same magnitude. When two comparisons have different nominal values, linking would be a challenge unless there is strong correlation between the two and the corresponding uncertainty is estimable. As an example, in Sec. 5 the methodology is applied to link the CCEM-K2 and SIM.EM-K2 key comparisons for instance at the 1 G Ω level.

## 2. Statistical Models for Interlaboratory Comparisons With Linear Trends

In some key comparisons, the measurand has a trend or a drift and thus, the measurements of the transport artifacts made by the participating NMIs will show trends. References [8] and [9] proposed statistical approaches to KCs with linear trends for a single and multiple artifacts, respectively. A recent paper, Zhang et al. [12] provided a generalized method, which can deal with the case when multiple NMIs measure the traveling artifacts more than one time and when the uncertainty structure is more general. Since [8] and [9] can be treated as special cases of [12], we will adopt the statistical model and notations in [12] for the comparisons.

We assume that *N* laboratories participated in the first key comparison, for example, a CIPM KC. We assume also that there were *P* artifacts traveling together and for each artifact, the *n*th laboratory (*n* = 1, ⋯, *N*) makes *J_n_* measurements with *J_n_* ≥ 1. For the *p*th artifact (*p* = 1, ⋯, *P*), the *j*th measurement (or the *j*th average of the measurements) made at the *n*th laboratory, *X_nj_* (*p*) is measured at the time *t_nj_* (*p*) (*j* = 1, ⋯, *J_n_*). As in [12], we assume a simple linear regression holds for all the measurements, i.e.,

(1) $$X_{nj}(p)=\alpha_{n}(p)+\beta (p)t_{nj}(p)+e_{nj}(p),$$

for *j* = 1, ⋯, *J_n_*, *n* = 1, ⋯, ***N***, and *p* = 1, ⋯, *P*, where for a fixed artifact the slopes of the trends for all *N* laboratories are the same, while we allow different intercepts for different laboratories. We further assume that for each laboratory, the random error in the measurement *X_nj_*(*p*) can be expressed as

(2) $$e_{nj}(p)=e_{nj,}{}_{A}(p)+(1-I_{n}(p))e_{nj,B}(p)+I_{n}(p)e_{n,B}(p),$$

where the indicator *I_n_*(*p*) = 1 when the errors *e_nj,B_*(*p*) are the same for all the measurements made by the *n*th laboratory, and *I_n_*(*p*) = 0 otherwise. The random components *e_nj,A_*(*p*) and (*e_n,B_*(*p*), *e_nj,B_*(*p*)) are statistically independent of each other with standard uncertainties of *σ_nj,A_*(*p*) and (*σ_n,B_*(*p*), *σ_nj,B_*(*p*)), which are the Type A and Type B evaluations of standard uncertainty, respectively. This indicates that the measurements of different artifacts (whether by the same or by different laboratories) are statistically independent, while the measurements for the same artifact, made at the same laboratory can be independent or not, depending on the indicator *I_n_*(*p*). Regarding the case for different artifacts measured by the same laboratory we understand that: (a) the errors quantified by the Type A uncertainty, i.e., *e_nj,A_*(*p*), are statistically independent; (b) the errors quantified by the Type B uncertainty, i.e., *e_nj,B_*(*p*) or *e_n,B_*(*p*) definitely have some correlation; (c) since not all artifacts are created equal and even when the metrologists make every effort to measure artifacts in as “correlated” a way as possible, there is still a random component. Thus, we think it is reasonable to assume that measurements of different artifacts (whether by the same or by different laboratories) are statistically independent. From (2), when *I_n_*(*p*) = 1, the Type B uncertainties are the same for all the measurements made on the *p*th artifact by the *n*th laboratory. On the contrary when *I_n_*(*p*) = 0, the Type B uncertainties may be not the same for all the measurements made by the *n*th laboratory. Without loss of generality, we assume that the pilot laboratory is the first one among all *P* laboratories with *J*_1_ > 1.

From Eqs. (10) and (11) in [12], the generalized least-squares estimators of *a_n_*(*p*) and *β* (*p*), which are the best linear unbiased estimators of these parameters, are given by

(3) $${\hat{\alpha }}_{n}(p)=X_{n}(p)-\hat{\beta }(p)t_{n}(p),\;n=1,\cdots ,N,$$

(4) $$\hat{\beta }(p)=\frac{\sum_{n=1}^{N}\sum_{j=1}^{J_{n}}\frac{\left(t_{nj}(p)-t_{n}(p))(X_{nj}(p)-X_{n}(p)\right)}{\sigma_{nj,A}^{2}(p)+(1-I_{n}(p))\sigma_{nj,B}^{2}(p)}}{\sum_{n=1}^{N}\sum_{j=1}^{J_{n}}\frac{{\left(t_{nj}(p)-t_{n}(p)\right)}^{2}}{\sigma_{nj,A}^{2}(p)+(1-I_{n}(p))\sigma_{nj,B}^{2}(p)}},$$

where

(5) $$t_{n}(p)=\sum_{j=1}^{J_{n}}w_{nj}(p)t_{nj}(p),\;X_{n}(p)=\sum_{j=1}^{J_{n}}w_{nj}(p)X_{nj}(p)$$

are weighted means of {*t_nj_*(*p*)} and {*X_nj_*(*p*)} with

(6) $$w_{nj}(p)=\frac{1/(\sigma_{nj,A}^{2}(p)+(1-I_{n}(p))\sigma_{nj,B}^{2}(p))}{\sum_{j=1}^{J_{n}}1/(\sigma_{nj,A}^{2}(p)+(1-I_{n}(p))\sigma_{nj,B}^{2}(p))},$$

respectively. From (5) and (6), the corresponding uncertainty for *X_n_*(*p*), *u_n_*(*p*), for the *p*th artifact in the *n*th laboratory is given by

(7) $$u_{n}^{2}(p)=\frac{1}{\begin{array}{l} \sum_{j=1}^{J_{n}}1/(\sigma_{nj,A}^{2}(p)+(1-I_{n}(p))\sigma_{nj,B}^{2}(p))\\ +I_{n}(p)\sigma_{n,B}^{2}(p). \end{array}}$$

As discussed in [8], [9], and [12], in cases with trends, the KCRV is time-dependent. As in [12], for a fixed set of weights *v* = {*v_p_*}, and at an optimal time

(8) $$t^{*}(p)=\sum_{n=1}^{N}\omega_{n}^{*}(\nu )t_{n}(p),\;p=1,\cdots ,P$$

with

(9) $$\omega_{n}^{*}(\nu )=\frac{1/\sum_{p=1}^{P}\nu_{p}^{2}u_{n}^{2}(p)}{\sum_{i=1}^{N}[1/\sum_{p=1}^{P}\nu_{p}^{2}u_{i}^{2}(p)]},$$

the corresponding optimal KCRV is given by

(10) $${\text{KCRV}}_{{\vec{t}}^{*}}=\sum_{n=1}^{N}\omega_{n}^{*}(\nu )\sum_{p=1}^{P}\nu_{p}X_{n}(p),$$

where ${\vec{t}}^{*}=(t^{*}(1),\cdots ,t^{*}(p),\cdots ,t^{*}(p))$. The corresponding uncertainty is given by

(11) $$u_{{\text{KCRV}}_{{\vec{t}}^{*}}}^{2}=\frac{1}{\sum_{n=1}^{N}[1/\sum_{p=1}^{P}\nu_{p}^{2}u_{n}^{2}(p)]}.$$

In practice, a choice of *v_p_* can be formed by the “mean-square residuals” for the *p*th regression line for the pilot libratory, i.e.,

(12) $$v_{p}=\frac{1/\rho^{2}(p)}{\sum_{i=1}^{P}1/\rho^{2}(i)},$$

where

(13) $$\rho^{2}(p)=\frac{\sum_{j=1}^{J_{1}}{\left(X_{1j}(p)-{\hat{\alpha }}_{1}(p)-\hat{\beta }(p)t_{1j}(p)\right)}^{2}}{J_{1}-2}.$$

In [8], the degree of equivalence of one laboratory with respect to the KCRV at some time *t* is defined as the difference between the predicted value of that laboratory based on the corresponding regression and the KCRV at *t*. From [9] and [12], for the first comparison, the degree of equivalence of the *n*th laboratory with respect to the KCRV $\vec{t}={\vec{t}}^{*}$ in (10) is the difference between a weighted mean of the predicted values of that laboratory for all artifacts and the corresponding regressions and the KCRV at $\vec{t}={\vec{t}}^{*}$. It is given by,

(14) $$D_{n,\text{KCRV}}=\sum_{p=1}^{P}\nu_{p}({\hat{\alpha }}_{n}(p)+\hat{\beta }(p)t^{*}(p))-{\text{KCRV}}_{{\vec{t}}^{*}}.$$

for *n* = 1, ⋯, *N*. For simplicity, we drop the ${\vec{t}}^{*}$ in the notation of *D_n_*_,KCRV_. The uncertainty of *D_n_*_,KCRV_ is provided by Eq. (33) in [12].

For the second comparison, for example, an RMO key comparison, we assume that there were *M* laboratories participating and *Q* artifacts traveling together. We also adopt the same statistical assumptions and models for the second comparison as used in the first comparison. Where necessary, a′ will be used to distinguish quantities in the second comparison from the analogous quantities in the first comparison. Under these assumptions, the corresponding weighted means of time and measurements as in (5), are ${t^{\prime }}_{m}(q)$ and *Y_m_*(*q*) for *m* = 1, ⋯, *M* and *q* = 1, ⋯, *Q*. Similarly, corresponding key comparison reference value ${\text{KCR}V^{\prime }}_{{i^{\prime }}^{*}}$ is obtained using the methods previously outlined for the first comparison. Similar to the first comparison, the degree of equivalence of the laboratory with respect to ${\text{KCR}V^{\prime }}_{{i^{\prime }}^{*}}$ at the optimal $\vec{t}={{\vec{t}}^{\prime }}^{*}$ is then given by

(15) $${D^{\prime }}_{m,\text{KCR}V^{\prime }}=\sum_{q=1}^{Q}{\nu^{\prime }}_{q}[{{\hat{\alpha }}^{\prime }}_{m}(q)+{\hat{\beta }}^{\prime }(q){t^{\prime }}^{*}(q)]-{\text{KCR}V^{\prime }}_{{{\vec{t}}^{\prime }}^{*}}$$

for *m* = 1, ⋯, *M.* We also assume that the artifacts used in the first comparison are different from those used in the first comparison.

## 3. The Difference Between the Degrees of Equivalence of the Two Comparisons With Respect to Their KCRVs

In order to be able to link two comparisons, we must assume that *K* laboratories, called linking laboratories, participated in both comparisons. In the case of no trend, [7] proposed to use a weighted mean of the differences between the measurements in the two comparisons for each linking laboratory. On the other hand, [1] and [2] used a weighted mean of the differences between the degrees of equivalences of the national measurement standards with respect to the KCRVs of the two comparisons for each linking laboratory. It is clear that the difference between the degrees of equivalences of the national measurement standards with respect to the KCRVs of the two comparisons for a linking laboratory contains information not only about the difference of the measurements in the two comparisons for the same laboratory but also about the differences of the measurements of other laboratories through the two KCRVs. We think that the combined difference in the second approach used in [1] and [2] represents the difference between the two comparisons better and thus adopt it.

Without loss of generality, we assume that the first *K* laboratories in both comparisons are the linking laboratories. Namely, for the *p*th artifact (*p* = 1, ⋯, *P*), *X*_1_(*p*), *X*_2_(*p*), ⋯ *X_K_*(*p*), from (5) are the representative measurements from the linking laboratories in the first comparison while *Y*_1_ (*q*), *Y*_2_ (*q*), ⋯ *Y_K_* (*q*), *q* = 1, ⋯, *Q*, are from the linking laboratories for the *q*th artifact in the second comparison. Note that *K* < min(*M*,*N*). For the *k*th linking laboratory, as considered in [1] and [2] the difference between the two degrees of equivalence given in (14) and (15) is given by

(16) $$D_{k}=D_{k,\text{KCRV}}-{D^{\prime }}_{k,\text{KCR}V^{\prime }}$$

for *k* = 1, ⋯, *K*. Since the KCRV of a comparison in our case is time-dependent, for the chosen optimal time, *D_k_*_,KCRV_ is a relative quantity with respect to that KCRV. We treat *D_k_* as a realization of the difference between the degrees of equivalence of the two comparisons for the *k*th linking laboratory. We assume that *D_k_* is random as in the statistical model given by

$$D_{k}=D+\eta_{k},$$

where *D* is the true value of the difference between the degrees of equivalence of the two comparisons and the random error *η_k_*, *k* = 1, ⋯, *K*, corresponds to the *k*th linking laboratory with zero mean. We use a weighted mean of *D_k_* (*k* = 1, ⋯, *K*) to estimate *D*. Namely,

(17) $$\hat{D}=\sum_{k=1}^{K}\psi_{k}D_{k}.$$

We use the weights given by

(18) $$\psi_{k}=\frac{1/\mathrm{Var}[D_{k}]}{\sum_{j=1}^{K}1/\mathrm{Var}[D_{j}]}.$$

The quantity $\hat{D}$ will be used to estimate the differences between the degrees of equivalence of two laboratories of which one only participated in the CIPM KC and the second one only participated in the RMO KC or vice versa. Note that {*D_k_*} are correlated because *D_k,_*_KCRV_ and *D_j,_*_KCRV_ as well as ${D^{\prime }}_{k,\text{KCR}V^{\prime }}$ and ${D^{\prime }}_{j,\text{KCR}V^{\prime }}$ for any *k* ≠ *j* and *k*, *j* = 1, ⋯, *K*, are correlated. Thus, the variance of $\hat{D}$ with *Ψ_k_* given in (18) is not equal to $\frac{1}{\sum_{k=1}^{K}1/\mathrm{Var}[D_{k}]}$ as given, e.g., in [13] when {*D_k_*} are statistically independent from each other, and is calculated as follows:

(19) $$\mathrm{Var}[\hat{D}]=\sum_{k=1}^{K}\psi_{k}^{2}\mathrm{Var}[D_{k}]+\sum_{k\neq l,k,l=1}^{K}\psi_{k}\psi_{l}\text{Cov}[D_{k},D_{l}],$$

where

(20) $$\begin{array}{l} \mathrm{Var}[D_{k}]=\mathrm{Var}[D_{k,\text{KCRV}}-{D^{\prime }}_{k,\text{KCR}V^{\prime }}]\\ \;=\mathrm{Var}[D_{k,\text{KCRV}}]+\mathrm{Var}[{D^{\prime }}_{k,\text{KCR}V^{\prime }}] \end{array}$$

and

(21) $$\begin{array}{l} \text{Cov}[D_{k},D_{l}]=\text{Cov}[D_{k,\text{KCRV}}-{D^{\prime }}_{k,\text{KCR}V^{\prime }},D_{l,\text{KCRV}}-{D^{\prime }}_{l,\text{KCR}V^{\prime }}]\\ \;=\text{Cov}[D_{k,\text{KCRV}},D_{l,\text{KCRV}}]\\ \;+\text{Cov}[{D^{\prime }}_{k,\text{KCR}V^{\prime }},{D^{\prime }}_{l,\text{KCR}V^{\prime }}]. \end{array}$$

Equation 20 holds because $\text{Cov}[D_{k,\text{KCRV}},{D^{\prime }}_{k,\text{KCR}V^{\prime }}]=0$ due to the assumption that the measurements of different artifacts made by the same laboratory are statistically independent as discussed in Sec. 2. Equation (21) holds since $\text{Cov}[D_{k,\text{KCRV}},{D^{\prime }}_{l,\text{KCR}V^{\prime }}]=0$ and $\text{Cov}[{D^{\prime }}_{k,\text{KCR}V^{\prime }},D_{l,\text{KCRV}}]=0$ for any *k* ≠ *l*. From (19), (20), and (21), it follows that

(22) $$\begin{array}{l} \mathrm{Var}[\hat{D}]=\sum_{k=1}^{K}\psi_{k}^{2}\{\mathrm{Var}[D_{k,\text{KCRV}}]+\mathrm{Var}[{D^{\prime }}_{k,\text{KCR}V^{\prime }}]\}\\ \;+\sum_{k\neq l,k,l=1}^{K}\psi_{k}\psi_{l}\text{Cov}[D_{k,\text{KCRV}},D_{l,\text{KCRV}}].\\ \;+\sum_{k\neq l,k,l=1}^{K}\psi_{k}\psi_{l}\text{Cov}[{D^{\prime }}_{k,\text{KCR}V^{\prime }},{D^{\prime }}_{l,\text{KCR}V^{\prime }}] \end{array}$$

In (23), Var[*D_k_*_,KCRV_] and $\mathrm{Var}[{D^{\prime }}_{k,\text{KCR}V^{\prime }}]$ are obtained from the two comparisons based on Eq. (33) in [12]. The covariance Cov[*D_k_*_,KCRV_, *D_l,_*_KCRV_] for *k* ≠ *l*, which may not be provided as part of the reports on the comparisons, is given by

(23) $$\begin{array}{l} \text{Cov}[D_{k,\text{KCRV}},D_{l,\text{KCRV}}]\\ =\sum_{p=1}^{P}\nu_{p}^{2}\cdot \frac{t_{k}(p)t_{l}(p)-t^{*}(p)(t_{k}(p)+t_{l}(p))+t^{*2}(p)}{\sum_{n=1}^{N}\sum_{j=1}^{J_{n}}\frac{{\left(t_{nj}(p)-t_{n}(p)\right)}^{2}}{\sigma_{nj,A}^{2}(p)+(1-I_{n}(p))\sigma_{nj,B}^{2}(p)}}\\ \;-\mathrm{Var}[{\text{KCRV}}_{{\vec{t}}^{*}}]. \end{array}$$

The derivation of (23) is in Appendix. Similar to (23), for $\text{Cov}[{D^{\prime }}_{k,\text{KCR}V^{\prime }},{D^{\prime }}_{l,\text{KCR}V^{\prime }}]$ for *k* ≠ *l* is given by

(24) $$\begin{array}{l} \text{Cov}[{D^{\prime }}_{k,\text{KCR}V^{\prime }},{D^{\prime }}_{l,\text{KCR}V^{\prime }}]\\ =\sum_{q=1}^{Q}{\nu^{\prime }}_{q}^{2}\cdot \frac{{t^{\prime }}_{k}(q){t^{\prime }}_{l}(q)-{t^{\prime }}^{*}(q)({t^{\prime }}_{k}(q)+{t^{\prime }}_{l}(q))+{t^{\prime }}^{*2}(q)}{\sum_{m=1}^{M}\sum_{j=1}^{J_{m}}\frac{{\left({t^{\prime }}_{mj}(q)-{t^{\prime }}_{m}(q)\right)}^{2}}{{\sigma^{\prime }}_{mj,A}^{2}(q)+(1-I_{m}(q)){\sigma^{\prime }}_{mj,B}^{2}(q)}}\\ \;-\mathrm{Var}[{\text{KCR}V^{\prime }}_{{\vec{t}}^{*}}]. \end{array}$$

where *J_m_* is the number of measurements for each artifact made at the *m*th laboratory. From (22), (23), and (24), $\mathrm{Var}[\hat{D}]$ or equivalently, the uncertainty of the difference between the degrees of equivalence of the two comparisons can be calculated.

We now consider the case of one laboratory only participated in the second comparison, e.g., a RMO key comparison. We need to find the degree of equivalence of this laboratory with respect to the KCRV of the first comparison. Because the *m*th laboratory only participated in the second comparison, *m* > *K*. Thus, we use the estimator below (denoted by *D ^#^_m,_*_KCRV_) to estimate the degree of equivalence of the *m*th laboratory with respect to the KCRV for the first comparison **had this laboratory participated in the first comparison**,

(25) $$D_{m,\text{KCRV}}^{\#}=D_{m,\text{KCRV}}^{\prime }+\hat{D}.$$

From (25),

(26) $$\begin{array}{l} \mathrm{Var}[D_{m,\text{KCRV}}^{\#}]=\mathrm{Var}[{D^{\prime }}_{m,\text{KCRV'}}]+\mathrm{Var}[\hat{D}]\\ \;+2\text{Cov}[{D^{\prime }}_{m,\text{KCRV'}},\hat{D}]. \end{array}$$

From (17) when *m* > *K*,

(27) $$\begin{array}{l} \text{Cov}[{D^{\prime }}_{m,\text{KCR}V^{\prime }},\hat{D}]\\ =\sum_{k=1}^{K}\psi_{k}\text{Cov}[{D^{\prime }}_{m,\text{KCR}V^{\prime }},D_{k}]\\ =\sum_{k=1}^{K}\psi_{k}\text{Cov}[{D^{\prime }}_{m,\text{KCR}V^{\prime }},D_{k,\text{KCRV}}-{D^{\prime }}_{k,\text{KCR}V^{\prime }}]\\ =\sum_{k=1}^{K}\psi_{k}\text{Cov}[{D^{\prime }}_{m,\text{KCR}V^{\prime }},{D^{\prime }}_{k,\text{KCR}V^{\prime }}]. \end{array}$$

Thus, from (26) and (27) for *m* > *K*,

(28) $$\begin{array}{l} \mathrm{Var}[D_{m,\text{KCRV}}^{\#}]=\mathrm{Var}[{D^{\prime }}_{m,\text{KCR}V^{\prime }}]+\mathrm{Var}[\hat{D}]+\\ \;2\sum_{k=1}^{K}\psi_{k}\text{Cov}[{D^{\prime }}_{m,\text{KCR}V^{\prime }},{D^{\prime }}_{k,\text{KCR}V^{\prime }}], \end{array}$$

where $\mathrm{Var}[{D^{\prime }}_{m,\text{KCR}V^{\prime }}]$ and $\text{Cov}[{D^{\prime }}_{m,\text{KCR}V^{\prime }},{D^{\prime }}_{k,\text{KCR}V^{\prime }}]$ are estimated from the data for the second comparison. The square root of $\mathrm{Var}[D_{m,\text{KCRV}}^{\#}]$ is the standard uncertainty of $D_{m,\text{KCRV}}^{\#}$.

## 4. Pair-Wise Comparisons—Degrees of Equivalence of Pairs of National Measurement Standards

These degrees of equivalence are for any pair of two different laboratories in the two key comparisons.

1. For any two laboratories participating in the first comparison, e.g., the CIPM KC (regardless of whether they participated in the RMO KC or not), their degrees of equivalence and the corresponding uncertainties are based on the results from the first comparison.
2. If two laboratories participated only in the second comparison or one laboratory participated in both comparisons and the second one only participated in the second comparison, then their degree of equivalence is the corresponding one in the second comparison with its uncertainty.
3. In the case that one laboratory only participated in the first comparison and the second laboratory only participated in the second comparison, the degree of equivalence between the *n*th laboratory (*n* > *K*), which participated only in the first comparison and the *m*th laboratory (*m* > *K*), which participated only in the second comparison, is estimated from (25) and given by
  (29) $$\begin{array}{l} D_{nm}^{\#}=D_{n,\text{KCRV}}-D_{m,\text{KCRV}}^{\#}\\ \;=D_{n,\text{KCRV}}-{D^{\prime }}_{m,\text{KCR}V^{\prime }}-\hat{D}. \end{array}$$

From (17), the uncertainty of $D_{nm}^{\#}$ for *n* = *K*+ 1, …, *N* and *m* = *K* + 1,…, *M* is given by

(30) $$\begin{array}{l} \mathrm{Var}[D_{nm}^{\#}]=\mathrm{Var}[D_{n,\text{KCRV}}]+\mathrm{Var}[{D^{\prime }}_{m,\text{KCR}V^{\prime }}]\\ \;+\mathrm{Var}[\hat{D}]-2\text{Cov}[D_{n,\text{KCRV}},\hat{D}]\\ \;+2\text{Cov}[{D^{\prime }}_{m,\text{KCR}V^{\prime }},\hat{D}]\\ \;-2\text{Cov}[D_{n,\text{KCRV}},{D^{\prime }}_{m,\text{KCR}V^{\prime }}]\\ \;=\mathrm{Var}[D_{n,\text{KCRV}}]+\mathrm{Var}[{D^{\prime }}_{m,\text{KCR}V^{\prime }}]\\ \;+\mathrm{Var}[\hat{D}]-2\text{Cov}[D_{n,\text{KCRV}},\hat{D}]\\ \;+2\text{Cov}[{D^{\prime }}_{m,\text{KCR}V^{\prime }},\hat{D}]. \end{array}$$

In the first equality, $\text{Cov}[D_{n,\text{KCRV}},{D^{\prime }}_{m,\text{KCR}V^{\prime }}]=0$ because $D_{n,\text{KCR}V^{\prime }}$ and ${D^{\prime }}_{m,\text{KCR}V^{\prime }}$ are for two separate comparisons using different artifacts and thus they are statistically independent from each other as discussed in Sec. 2. The first two terms in the second equality in (30) are obtained from the two comparisons, respectively. The variance of $\hat{D}$ is given by (22) to (24). The term $\text{Cov}[{D^{\prime }}_{m,\text{KCR}V^{\prime }},\hat{D}]$ is given by (27). Similar to (27) for *n* > *K*

(31) $$\begin{array}{l} \text{Cov}[D_{n,\text{KCRV}},\hat{D}]\\ =\sum_{k=1}^{K}\psi_{k}\text{Cov}[D_{n,\text{KCRV}},D_{k,\text{KCRV}}]. \end{array}$$

Therefore, the uncertainty of $D_{nm}^{\#}$ for *n*, *m* > *k* is obtained from (30) and (31).

This linking methodology is based on the model and the approach proposed in [12] for the case of trend. Although the mathematical derivations for the linking as well as the results from [12] seem complicate, the calculations are straightforward. We used MATLAB^1^ [14] to implement the method for SIM.EM-K1, SIM.EM-K2, and SIM.EM-S6 comparisons [15] as well for the example in Sec. 5.

## 5. Linking the CCEM-K2 and SIM.EM-K2 Comparisons

To illustrate this linking approach, we applied it to the CCEM-K2 key comparison for resistance at the level of 1 G Ω and the SIM.EM-K2 key comparison for resistance at the same level. From 2006 to 2007, the Working Group for Electricity and Magnetism of the Inter-American Metrology System (SIM) conducted the key and supplementary comparisons SIM.EM-K1-K2-S6 to provide the first internationally recognized comparisons of precision resistance measurements for nations of the western hemisphere. Six NMIs participated in the comparisons. The National Institute of Standards and Technology (NIST) provided the comparison standards and acted as the pilot laboratory. Two NIST film-type resistors were used as traveling standards. Over the course of the comparison, the two traveling standards were measured at the pilot laboratory, NIST, during five time periods. For each period, an average value of the dates when the measurements were made was calculated and called the mean date of measurement. In the SIM.EM-K2 comparison, each of the five non-pilot laboratories made measurements during two separate time periods except one which only measured at one time period. An uncertainty budget that includes the Type A and Type B evaluations of uncertainties for each NMI’s measurement process was also reported.

**Fig. 1:**
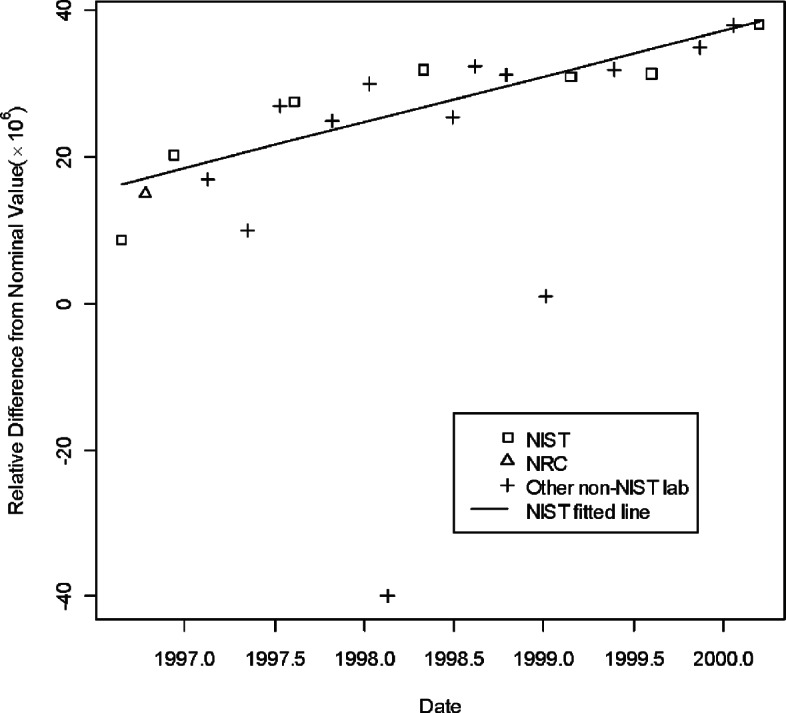
Measurements of 1 G Ω standard S/N 9101 by all participants in CCEM-K2 and the regression line based on NIST measurements.

**Fig. 2:**
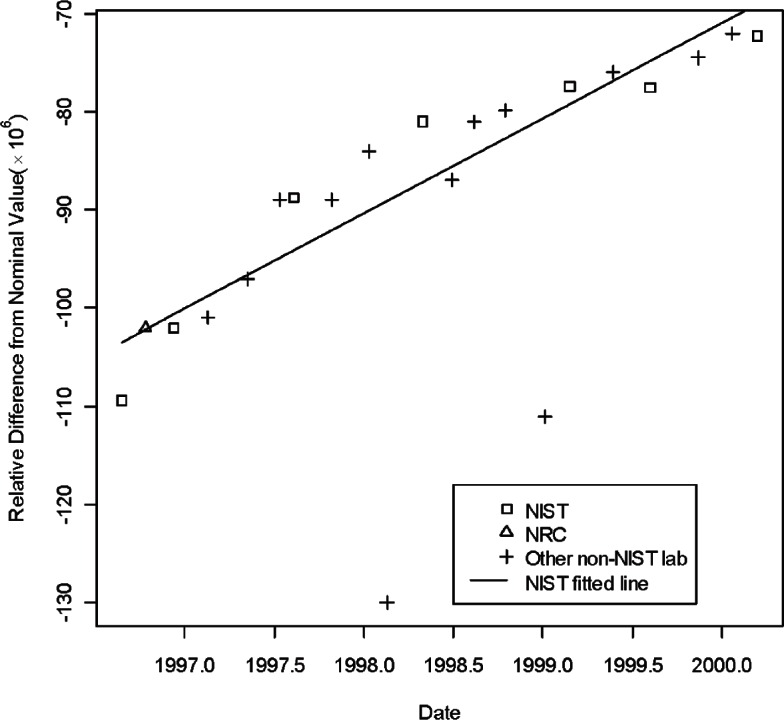
Measurements of 1 G Ω standard S/N 9102 by all participants in CCEM-K2 and the regression line based on NIST measurements.

**Fig. 3:**
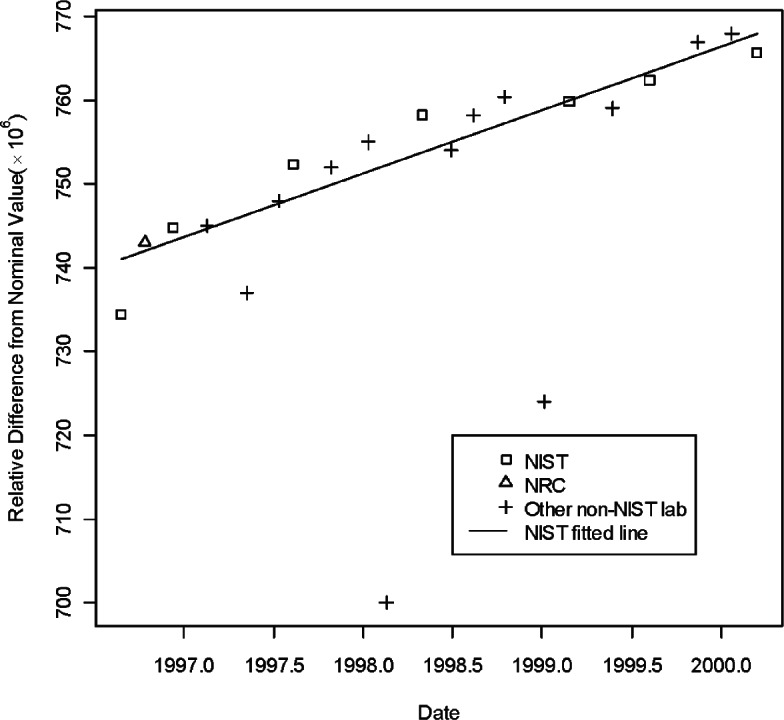
Measurements of 1 G Ω standard S/N 9106 by all participants in CCEM-K2 and the regression line based on NIST measurements.

Table 1 lists the information describing the CCEM-K2 results taken from Table 5 of [16]. In the table, the listed resistance measurements are relative deviations from the norminal value. Namely, in the table the entries for the three artifacts, S/N HR 9101, S/N HR 9102, and S/N HR 9106 are expressed as (measurement value— 1 G Ω)× 10^6^/1 G Ω. NIST was the pilot laboratory and the only laboratory to make multiple measurements in seven time periods. Figures 1 to 3 show the three regression lines corresponding to the measurements of the three traveling standards made by NIST, the pilot laboratory. The figures also show the measurements made by all participating laboratories.

**Fig. 4:**
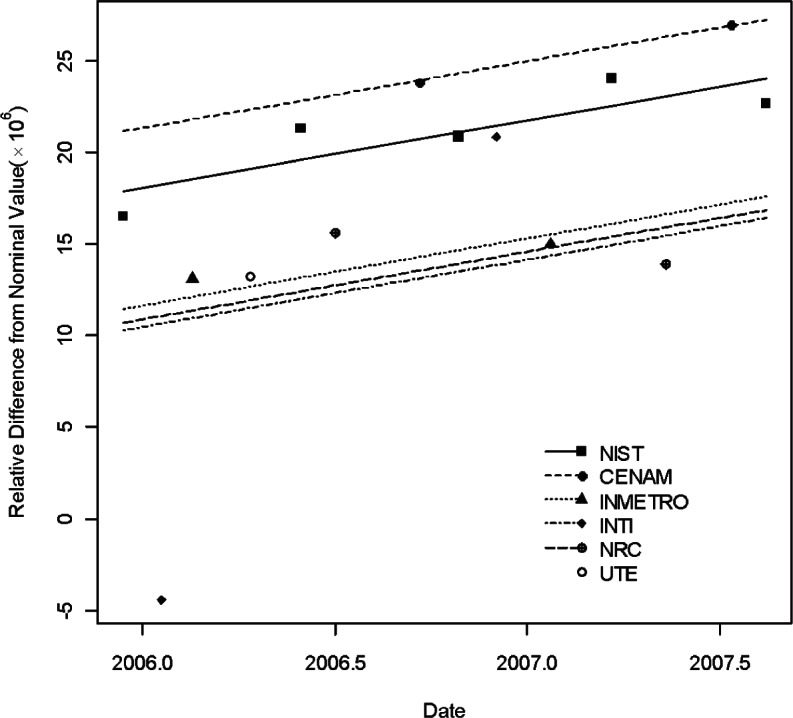
Measurements of 1 G Ω standard S/N 9104 by all participants in SIM.EM-K2 and the regression lines.

**Fig. 5:**
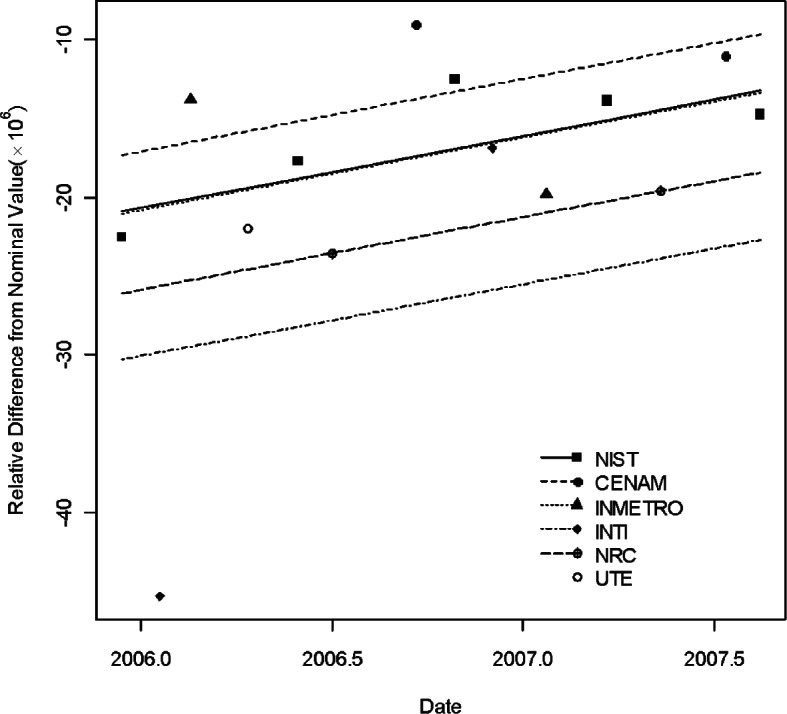
Measurements of 1 G Ω standard S/N 9105 by all participants in SIM.EM-K2 and the regression lines.

Tables 2 and 3 list the information for the two traveling standards used in the SIM.EM-K2 comparison [15]. Figures 4 and 5 show the five regression lines corresponding to the five laboratories each with two or more measurements. These two figures were published in [12]. Table 4 lists the degrees of equivalence of national measurement standards with respect to the KCRV (× 10^6^) (KCRV = 301.0 at t^*^ = 1998.8) and the associated uncertianties from CCEM-K2. The results were calculated based on the statistical analysis from [12]. Table 5 lists the degrees of equivalence of national measurement standards with respect to the KCRV and the associated uncertianties from SIM.EM-K2 comparison based on [12]. The other results can be found in [12].

**Fig. 6:**
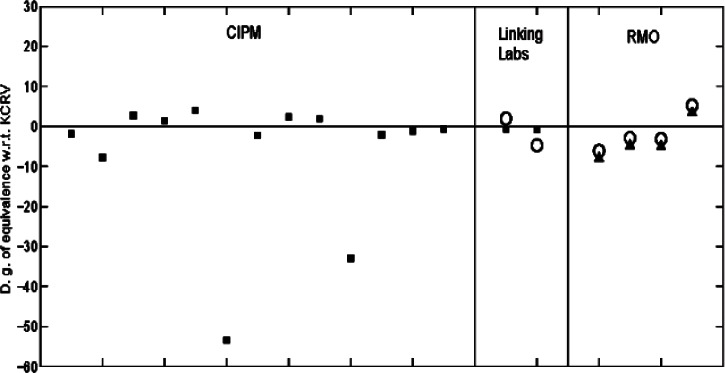
The degrees of equivalence of national measurement standards with respect to the KCRVs (× 10^6^) for the two comparisons. The linking laboratories are NIST and NRC. The solid squares represent the degrees of equivalence of the CIPM national measurement standards with respect to the CIPM KCRV for CCEM-K2 while the open circles represent the degrees of equivalence of the RMO national measurement standards with respect to the RMO KCRV for SIM.EM-K2. The solid triangles represent the degrees of equivalence of the RMO national measurement standards with respect to the CIPM KCRV for the four non-linking laboratories in the RMO comparison.

There were two linking laboratories: NIST and the National Research Council (NRC) of Canada. Figure 6 shows the degrees of equivalence with respect to the KCRVs for the two comparisons as listed in Tables 4 and 5. From (16) and (20), the *D_k_* for *k* = 1,2 and their associate standard uncertainties corresponding to NIST and NRC were calculated. From (17) to (24), $\hat{D}$, the weighted mean of the *D_k_* with weights based on (18) and its standard uncertainty (× 10^6^) were calculated to the values $\hat{D}=-1.87$ and *u_D_* = 2.86. Note that the covariances of {*D_n_*_,KCRV_} and $\left\{{D^{\prime }}_{m,\text{KCR}V^{\prime }}\right\}$ were not provided by the reports for CCEM-K2 and SIM.EM-K2. Instead, these terms, which are necessary for calculating the uncertainties of *D^^^* and other terms, were calculated using (23) and (24). For the four remaining NMIs in the SIM.EM-K2 comparison, which did not participate in CCEM-K2, the degrees of equivalence of their national standards with respect to the KCRV of CCEM-K2 comparison were calculated using (25). Their corresponding standard uncertainties were calculated from (28) and are listed in Table 6.

Thirteen NMIs participated in the CCEM-K2 comparison but did not participate in the SIM.EM comparison. Similarly, four NMIs participated in the SIM.EM-K2 comparison but did not participate in the CCEM-K2 comparison. Figure 6 shows the degrees of equivalenceof the national measurement standards with respect to the KCRVs for the two comparisons. In Fig. 6, the solid squares represent the degrees of equivalence of the CIPM national measurement standards with respect to the CIPM KCRV, i.e., *D_n_*_,KCRV_ (*n* = 1,…, 15) for CCEM-K2 while the open circles represent the degrees of equivalence of RMO national measurement standards with respect to the RMO KCRV, i.e., ${D^{\prime }}_{m,\text{KCR}V^{\prime }}(m=1,\ldots ,6)$ for SIM.EM-K2. For the four non-linking laboratories in the RMO comparison, the degrees of equivalence of the RMO national measurement standards with respect to the CIPM KCRV were calculated by (25) and represented by solid triangles. Pair-wise comparisons between the NMIs of these two groups, i.e., their degrees of equivalence and their associate standard uncertainties were calculated using (29) to (31) and are listed in Table 7.

## 6. Conclusions

Statistical approaches have been developed recently to deal with interlaboratory comparisons with linear trends. In this paper, a statistical analysis is proposed to link two interlaboratory key comparisons, where both have the same nominal value or values of a same magnitude and both show linear trends. The degrees of equivalence, either with respect to the KCRV of the CIPM KC for those laboratories that did not participate in the CIPM KC or between any two laboratories that participated in only one of the two comparisons are obtained with their associated uncertainties.

## Figures and Tables

**Table 1:** Results of the comparison CCEM-K2 at 1 G Ω, expressed as relative difference from the nominal value (10^6^ × Relative difference from nominal value)

Laboratory	Mean Date of Measurements	S/N HR9101	S/N HR9102	S/N HR9106	10^6^ × Type A Standard Uncertainy	10^6^ × Type B Standard Uncertainy
NIST	1996-08-24	8.7	−109.4	734.4	2.0	4.6
NRC	1996-10-11	15	−102	743	3.8	9.2
NIST	1996-12-08	20.3	−102.0	744.8	2.0	4.6
BNM-LCIE	1997-02-18	17	−101	745	2.9	8.4
NPL	1997-05-08	10	−97	737	1.5	4.8
PTB	1997-07-12	27	−89	748	2.5	5.8
NIST	1997-08-12	27.6	−88.8	752.3	2.0	4.6
CSIRO-NML	1997-10-25	25	−89	752	2.0	33
MSL	1998-01-11	30	−84	755	0.9	2.2
CSIR-NML	1998-02-15	−40	−130	700	50	289
NIST	1998-04-29	31.9	−81.0	758.2	2.0	4.6
SP	1998-06-27	25.5	−86.9	754.0	0.5	4.4
OFMET	1998-08-15	32.4	−81.0	758.2	4.2	10.8
IEN	1998-10-17	31.2	−79.9	760.4	3.5	9.1
NMi-VSL	1999-01-03	1	−111	724	8.0	17
NIST	1999-02-22	31.0	−77.4	759.9	2.0	4.6
KRISS	1999-05-23	32	−76	759	0.7	5.6
NIST	1999-08-07	31.4	−77.5	762.4	2.0	4.6
NIM	1999-11-14	35.0	−74.5	766.9	1.0	3.1
VNIIM	2000-01-23	38	−72	768	1.0	2.3
NIST	2000-03-13	38.1	−72.3	765.6	2.0	4.6

**Table 2:** Results at 1 G Ω, expressed as relative difference from the nominal value (10^6^ × Relative difference from nominal value) for Standard HR9104 in SIM.EM.K2

Laboratory	Mean Date of Measurements	Reported Resistance	10^6^ × Type A Standard Uncertainy	10^6^ × Type B Standard Uncertainy
NIST	26-Dec-2005	16.53	0.86	2.69
INTI	19-Jan-2006	−4.42	8.00	7.32
INMETRO	18-Feb-2006	13.10	7.00	6.09
UTE	12-Apr-2006	13.20	2.32	22.12
NIST	1-Jun-2006	21.34	0.88	2.69
NRC	12-Aug-2006	15.60	1.33	12.50
CENAM	20-Sep-2006	23.80	1.00	17.58
NIST	26-Oct-2006	20.89	1.35	2.69
INTI	3-Dec-2006	20.83	0.60	7.35
INMETRO	21-Jan-2007	15.00	3.31	6.12
UTE	Laboratory did not participate in this round of the comparison			
NIST	23-Mar-2007	24.08	1.12	2.69
NRC	11-May-2007	13.90	0.43	10.58
CENAM	13-Jul-2007	27.00	0.78	10.09
NIST	15-Aug-2007	22.72	0.92	2.69

**Table 3:** Results at 1 G Ω, expressed as relative difference from the nominal value (10^6^ × Relative difference from nominal value) for Standard HR9105 in SIM.EM.K2

Laboratory	Mean Date of Measurements	Reported Resistance	10^6^ × Type A Standard Uncertainy	10^6^ × Type B Standard Uncertainy
NIST	26-Dec-2005	−22.53	1.39	2.69
INTI	19-Jan-2006	−45.35	8.00	9.41
INMETRO	18-Feb-2006	−13.80	6.80	6.98
UTE	12-Apr-2006	−22.00	1.47	22.12
NIST	1-Jun-2006	−17.69	1.65	2.69
NRC	12-Aug-2006	−23.60	1.58	12.58
CENAM	20-Sep-2006	−9.00	2.00	23.00
NIST	26-Oct-2006	−12.48	2.11	2.69
INTI	3-Dec-2006	−16.88	0.80	9.39
INMETRO	21-Jan-2007	−19.80	3.89	6.51
UTE	Laboratory did not participate in this round of the comparison			
NIST	23-Mar-2007	−13.86	2.11	2.69
NRC	11-May-2007	−19.60	0.75	10.58
CENAM	13-Jul-2007	−11.00	1.40	10.17
NIST	15-Aug-2007	−14.73	1.31	2.69

**Table 4:** The degrees of equivalence (× 10^6^) of national measurement standards with respect to KCRV and their standard uncertainties (× 10^6^) in CCEM-K2 for 1 G Ω

	NIST	NRC	BNM- LCIE	NPL	PTB	CSIRO- NML	MSL	CSIR-.NML
*D_i_*_,KCRV_	− 0.68	− 0.80	− 1.84	− 7.73	− 2.69	1.51	4.03	−53.47
$u_{D_{i,\text{KCRV}}}$	2.67	6.03	5.35	3.10	3.79	19.39	1.36	172.00

	SP	OFMET	IEN	NMi- VSL	KRISS	NIM	VNIM
*D_i_*_,KCRV_	− 2.15	2.31	1.88	−32.90	− 2.03	− 1.20	− 0.63
$u_{D_{i,\text{KCRV}}}$	2.51	6.76	5.67	11.00	3.26	1.97	1.63

**Table 5:** The degrees of equivalence (× 10^6^) of national measurement standards with respect to KCRV and their standard uncertainties (× 10^6^) in SIM.EM-K2 for 1 G Ω

	NIST	INTI	INMETRO	UTE	NRC	CENAM
${D^{\prime }}_{i,\text{KCR}V^{\prime }}$	1.94	−6.11	−2.92	− 3.14	− 4.72	5.28
$u_{{D^{\prime }}_{i,\text{KCR}V^{\prime }}}$	1.36	4.65	4.11	17.53	6.19	6.80

**Table 6:** The degrees of equivalence (× 10^6^) of four SIM national measurement standards with respect to KCRV from CCEM-K2 and their standard uncertainties (× 10^6^) for 1 G Ω

	INTI	INMETRO	UTE	CENAM
$D_{m,\text{KCRV}}^{\#}$	−7.97	−4.78	− 5.00	3.42
$u_{D_{m,\text{KCRV}}^{\#}}$	4.92	4.41	17.60	6.98

**Table 7:** The degrees of equivalence (×10^6^) of pairs of national measurement standards $D_{nm}^{\#}$ and their standard uncertainties $u_{D_{nm}^{\#}}(\times 10^{6})$ (in parentheses below the degrees of equivalence)

	BNM-LCIE	NPL	PTB	CSIRO-NML	MSL	CSIR-NML
INTI	6.13 (7.23)	0.24 (5.79)	10.66 (6.20)	9.48 (20.01)	12.00 (5.14)	− 45.50 (172.08)
INMETRO	2.93 (6.90)	−2.95 (5.37)	7.47 (5.80)	6.29 (19.89)	8.81 (4.65)	− 48.70 (172.06)
UTE	3.16 (18.38)	−2.73 (17.86)	7.69 (18.00)	6.51 (26.19)	9.03 (17.66)	− 48.47 (172.91)
CENAM	−5.26 (8.77)	−11.15 (7.62)	−0.73 (7.94)	−1.91 (20.62)	0.61 (7.14)	− 56.89 (172.15)

	SP	OFMET	IEN	NMi-VSL	KRISS	NIM	VNIIM
INTI	5.82 (5.59)	10.28 (8.41)	9.85 (7.57)	−24.93 (12.10)	5.94 (6.03)	6.78 (5.48)	7.34 (5.38)
INMETRO	2.63 (5.14)	7.09 (8.12)	6.65 (7.25)	−28.13 (11.90)	2.75 (5.62)	3.58 (5.02)	4.15 (4.92)
UTE	2.86 (17.80)	7.31 (18.88)	6.88 (18.52)	−27.90 (20.78)	2.97 (17.94)	3.81 (17.76)	4.38 (17.74)
CENAM	−5.56 (7.47)	−1.11 (9.76)	−1.54 (9.05)	−36.32 (13.07)	−5.45 (7.81)	−4.61 (7.39)	−4.04 (7.32)

## Footnotes

1 Certain commercial equipment, instruments, or materials are identified in this paper to foster understanding. Such identification does not imply recommendation or endorsement by the National Institute of Standards and Technology, nor does it imply that the materials or equipment identified are necessarily the best available for the purpose.

## 7. Appendix

The derivation of Cov[*D_k_*_,KCRV_, *D_l_*_,KCRV_] when *k* ≠ *l.*

From (14)

(A.1) $$\begin{array}{l} \text{Cov}[D_{k,\text{KCRV}},D_{l,\text{KCRV}}]\\ =\text{Cov}[\sum_{p=1}^{P}\nu_{p}[{\hat{\alpha }}_{k}(p)+\hat{\beta }(p)t^{*}(p)]-{\text{KCRV}}_{{\vec{t}}^{*}},\sum_{p=1}^{P}\nu_{p}[{\hat{\alpha }}_{l}(p)+\hat{\beta }(p)t^{*}(p)]-{\text{KCRV}}_{{\vec{t}}^{*}}]\\ =\text{Cov}[\sum_{p=1}^{P}\nu_{p}[{\hat{\alpha }}_{k}(p)+\hat{\beta }(p)t^{*}(p)],\sum_{p=1}^{P}\nu_{p}[{\hat{\alpha }}_{l}(p)+\hat{\beta }(p)t^{*}(p)]]+{\mathrm{Var}[\text{KCRV}}_{{\vec{t}}^{*}}]\\ -\text{Cov}[\sum_{p=1}^{P}\nu_{p}[{\hat{\alpha }}_{k}(p)+\hat{\beta }(p)t^{*}(p)],{\text{KCRV}}_{{\vec{t}}^{*}}]-{\text{Cov[KCRV}}_{{\vec{t}}^{*}},\sum_{p=1}^{P}\nu_{p}[{\hat{\alpha }}_{l}(p)+\hat{\beta }(p)t^{*}(p)]]\\ =\sum_{p=1}^{P}\nu_{p}^{2}\text{Cov}[{\hat{\alpha }}_{k}(p)+\hat{\beta }(p)t^{*}(p),{\hat{\alpha }}_{l}(p)+\hat{\beta }(p)t^{*}(p)]+{\mathrm{Var}[\text{KCRV}}_{{\vec{t}}^{*}}]\\ -\text{Cov}\sum_{p=1}^{P}\nu_{p}[{\hat{\alpha }}_{k}(p)+\hat{\beta }(p)t^{*}(p)],{\text{KCRV}}_{{\vec{t}}^{*}}]\\ -{\text{Cov[KCRV}}_{{\vec{t}}^{*}},\sum_{p=1}^{P}\nu_{p}[{\hat{\alpha }}_{l}(p)+\hat{\beta }(p)t^{*}(p)]] \end{array}$$

The third equality holds because the measurements from any two different artifacts are statistically independent from each other.

When *k* ≠ *l*,

(A.2) $$\begin{array}{l} \text{Cov}[{\hat{\alpha }}_{k}(p)+\hat{\beta }(p)t^{*}(p),{\hat{\alpha }}_{l}(p)+\hat{\beta }(p)t^{*}(p)]\\ =\text{Cov}[{\hat{\alpha }}_{k}(p),{\hat{\alpha }}_{l}(p)]+t^{*}(p)\text{Cov}[\hat{\beta }(p),{\hat{\alpha }}_{l}(p)]\\ +t^{*}(p)\text{Cov}[{\hat{\alpha }}_{k}(p),\hat{\beta }(p)]+t^{*2}(p)\mathrm{Var}[\hat{\beta }(p)]\\ =\frac{t_{k}(p)t_{l}(p)}{\sum_{n=1}^{N}\sum_{j=1}^{J_{n}}\frac{{\left(t_{nj}(p)-t_{n}(p)\right)}^{2}}{\sigma_{nj,A}^{2}(p)+(1-I_{n}(p))\sigma_{nj,B}^{2}(p)}}-\frac{t^{*}(p)t_{l}(p)}{\sum_{n=1}^{N}\sum_{j=1}^{J_{n}}\frac{{\left(t_{nj}(p)-t_{n}(p)\right)}^{2}}{\sigma_{nj,A}^{2}(p)+(1-I_{n}(p))\sigma_{nj,B}^{2}(p)}}\\ -\frac{t^{*}(p)t_{k}(p)}{\sum_{n=1}^{N}\sum_{j=1}^{J_{n}}\frac{{\left(t_{nj}(p)-t_{n}(p)\right)}^{2}}{\sigma_{nj,A}^{2}(p)+(1-I_{n}(p))\sigma_{nj,B}^{2}(p)}}+\frac{t^{*2}(p)}{\sum_{n=1}^{N}\sum_{j=1}^{J_{n}}\frac{{\left(t_{nj}(p)-t_{n}(p)\right)}^{2}}{\sigma_{nj,A}^{2}(p)+(1-I_{n}(p))\sigma_{nj,B}^{2}(p)}}\\ =\frac{t_{k}(p)t_{l}(p)-t^{*}(p)(t_{k}(p)+t_{l}(p))+t^{*2}(p)}{\sum_{n=1}^{N}\sum_{j=1}^{J_{n}}\frac{{\left(t_{nj}(p)-t_{n}(p)\right)}^{2}}{\sigma_{nj,A}^{2}(p)+(1-I_{n}(p))\sigma_{nj,B}^{2}(p)}.} \end{array}$$

The second equality is from Eqs. (14), (17), and (18) in [12]. For the third term of the last equality in (A.1),

(A.3) $$\begin{array}{l} \text{Cov}[\sum_{p=1}^{P}\nu_{p}[{\hat{\alpha }}_{k}(p)+\hat{\beta }(p)t^{*}(p)],{\text{KCRV}}_{{\vec{t}}^{*}}]\\ =\text{Cov}[\sum_{p=1}^{P}\nu_{p}[{\hat{\alpha }}_{k}(p)+\hat{\beta }(p)t^{*}(p)],\sum_{n=1}^{N}\omega_{n}^{*}(\nu )\sum_{p=1}^{P}\nu_{p}X_{n}(p)]\\ =\sum_{p=1}^{P}\nu_{p}^{2}\text{Cov}[{\hat{\alpha }}_{k}(p)+\hat{\beta }(p)t^{*}(p),\sum_{n=1}^{N}\omega_{n}^{*}(\nu )X_{n}(p)]. \end{array}$$

The first equality is from (10). We show that when *k* ≠ *n*,

$$\text{Cov}[{\hat{\alpha }}_{k}(p)+\hat{\beta }(p)t^{*}(p),X_{n}(p)]=0.$$

From (3), ${\hat{\alpha }}_{k}(p)=X_{n}(p)-\hat{\beta }(p)t_{k}(p)$. Thus,

(A.4) $$\begin{array}{l} \text{Cov}[{\hat{\alpha }}_{k}(p)+\hat{\beta }(p)t^{*}(p),X_{n}(p)\\ =\text{Cov}[X_{k}(p)+\hat{\beta }(p)(t^{*}(p)-t_{k}(p)),X_{n}(p)]\\ =(t^{*}(p)-t_{k}(p))\text{Cov}[\hat{\beta }(p),X_{n}(p)]\\ =0. \end{array}$$

The third equality holds because Cov [*X_k_* (*p*), *X_n_*(*p*)] = 0 and from Eq. (16) in [12],

(A.5) $$\text{Cov}[\hat{\beta }(p),X_{n}(p)]=0.$$

From (A.3) and (A.4)

(A.6) $$\begin{array}{l} \text{Cov}[\sum_{p=1}^{P}\nu_{p}[{\hat{\alpha }}_{k}(p)+\hat{\beta }(p)t^{*}(p)],{\text{KCRV}}_{{\vec{t}}^{*}}]\\ =\sum_{p=1}^{P}\nu_{p}^{2}\text{Cov}[{\hat{\alpha }}_{k}(p)+\hat{\beta }(p)t^{*}(p),\omega_{k}^{*}(\nu )X_{k}(p)]. \end{array}$$

Similar to (A.4),

(A.7) $$\begin{array}{l} \text{Cov}[{\hat{\alpha }}_{k}(p)+\hat{\beta }(p)t^{*}(p),X_{k}(p)]\\ =\text{Cov}[X_{k}(p)+\hat{\beta }(p)t^{*}(p)-t_{k}(p)),X_{k}(p)]\\ =\mathrm{Var}[X_{k}(p)]+(t^{*}(p)-t_{k}(p))\text{Cov}[\hat{\beta }(p),X_{k}(p)]\\ =\mathrm{Var}[X_{k}(p)]. \end{array}$$

From (A.6), and (A.7),

(A.8) $$\begin{array}{l} \text{Cov}[\sum_{p=1}^{P}\nu_{p}[{\hat{\alpha }}_{k}(p)+\hat{\beta }(p)t^{*}(p)],{\text{KCRV}}_{{\vec{t}}^{*}}]\\ =\omega_{k}^{*}(\nu )\sum_{p=1}^{P}\nu_{p}^{2}\mathrm{Var}[X_{k}(p)]\\ =\mathrm{Var}[{\text{KCRV}}_{{\vec{t}}^{*}}]. \end{array}$$

Since $\mathrm{Var}[X_{k}(p)]=u_{k}^{2}(p)$, from (9) and (11), the last equality holds. Similarly, for the fourth term in the last equality in (A.1),

(A.9) $$\begin{array}{l} \text{Cov}[{\text{KCRV}}_{{\vec{t}}^{*}},\sum_{p=1}^{P}\nu_{p}[{\hat{\alpha }}_{l}(p)+\hat{\beta }(p)t^{*}(p)]\\ =\omega_{l}^{*}(\nu )\sum_{p=1}^{P}\nu_{p}^{2}\mathrm{Var}[X_{l}(p)]\\ =\mathrm{Var}[{\text{KCRV}}_{{\vec{t}}^{*}}]. \end{array}$$

From (A.1), (A.2), (A.8), and (A.9), when *k* ≠ *l*,

(A.10) $$\begin{array}{l} \text{Cov}[D_{k,\text{KCRV}},D_{l,\text{KCRV}}]\\ =\sum_{p=1}^{P}\nu_{p}^{2}\frac{t_{k}(p)t_{l}(p)-t^{*}(p)(t_{k}(p)+t_{l}(p))+t^{*2}(p)}{\sum_{n=1}^{N}\sum_{j=1}^{J_{n}}\frac{{\left(t_{nj}(p)-t_{n}(p)\right)}^{2}}{\sigma_{nj,A}^{2}(p)+(1-I_{n}(p))\sigma_{nj,B}^{2}(p)}}-\mathrm{Var}[{\text{KCRV}}_{{\vec{t}}^{*}}] \end{array}$$

as given by (23).

## References

[1] Delahaye F, Witt T (2002). Linking the results of key comparions CCEM-K4 with the 10 pF results of EUROMET.EM-K4. Metrologia.

[2] Marullo Reedtz G, Cerri R (2004). Linking the results of key comparisons CCEM-K8 and EUROMET.EM-K8. Metrologia.

[3] Elster C, Link A, Wöger W (2003). Proposal for linking the results of CIPM and RMO key comparisons. Metrologia.

[4] Nielsen L (2002).

[5] Sutton CM (2004). Analysis and linking of international measurement comparisons. Metrologia.

[6] Kharitonov IA, Chunovkina AG (2006). Evaluation of regional key comparison data: two approaches for data processing. Metrologia.

[7] Decker EJ, Steele AG, Douglas RJ (2008). Measurement science and the linking of CIPM and regional key comparisons. Metrologia.

[8] Zhang NF, Liu HK, Strawderman WE, Sedransk N (2004). Statistical analysis of key comparisons with linear trends. Metrologia.

[9] Zhang NF, Strawderman WE, Liu HK, Sedransk N (2006). Statistical analysis for multiple artifact problem in key comparisons with linear trends. Metrologia.

[10] Elster C, Wöger W, Cox M (2005). Analysis of key comparison data: unstable travelling standards. Measurement Techniques.

[11] Stepanov AV (2007). General problems of metrology and measurement technique—Comparing algorithms for evaluating key comparisons with linear drift in the reference standard. Measurement Techniques.

[12] Zhang W, Zhang NF, Liu HK (2009). A generalized method for multiple artifacts problem in interlaboratory comparisons with linear trends. Metrologia.

[13] Zhang NF (2006). The uncertainty associated with the weighted mean of measurement data. Metrologia.

[14] Higham DJ, Higham NJ (2005). MATLAB Guide.

[15] Elmquist RE, Jarrett DG, Zhang NF (2009). RMO Comparison Final report 2006–2007, Resistance standards comparison between SIM laboratories, SIM.EM-K1, 1Ω, SIM.EM-K2, 1GΩ, SIM.EM-S6, 1MΩ. Metrologia.

[16] Dziuba RF, Jarrett DG (2002). Final Report on key Comparison CCEM-K2 of reisistance standards at 10MΩ and 1GΩ. Metrologia.

